# Case report: A case of novel homozygous LRBA variant induced by chromosomal segmental uniparental disomy - genetic and clinical insights

**DOI:** 10.3389/fimmu.2024.1351076

**Published:** 2024-03-05

**Authors:** Lihua Jiang, Sen Chen

**Affiliations:** Hematology Department, Tianjin Children’s Hospital (Children’s Hospital, Tianjin University), Tianjin Key Laboratory of Birth Defects for Prevention and Treatment, Tianjin, China

**Keywords:** LRBA gene variant, uniparental disomy, paternal origin, immune dysregulation, pancytopenia

## Abstract

**Objective:**

The study aims to report a rare case of a novel homozygous variant in the LRBA gene, originating from uniparental disomy of paternal origin. This case contributes new clinical data to the LRBA gene variant database.

**Methods:**

The study details the case of a 2-year-old child diagnosed in May 2023 at our center with a homozygous LRBA gene variant. Detailed clinical data of the patient were collected, including whole-exome sequencing of peripheral blood mononuclear cells, with parental genetic verification.

**Results:**

The child presented with recurrent respiratory infections and chronic neutropenia, progressing to pancytopenia. Imaging showed splenomegaly and enlarged lymph nodes in the axillary and abdominal regions. Peripheral blood lymphocyte count revealed reduced B cells and NK cells. Elevated cytokine levels of IFN-α and IFN-r were observed. Whole-exome sequencing revealed a nonsense homozygous variant in the LRBA gene, specifically c.2584C>T (p.Gln862Ter). The father exhibited a heterozygous variant at this locus, while no variant was found in the mother. Sample analysis indicated characteristics of uniparental disomy. According to the guidelines of the American College of Medical Genetics and Genomics (ACMG), this variant is preliminarily classified as “Likely pathogenic”. Currently, there are no reports in academic literature regarding this specific variant site.

**Conclusion:**

LRBA gene variants can lead to a rare inborn error of immunity disease. The c.2584C>T (p.Gln862Ter) variant in exon 22 of the LRBA gene is a newly identified pathogenic variant, and the homozygous variant caused by uniparental disomy is exceedingly rare. This case represents the second global report of an LRBA gene function loss due to uniparental disomy abnormalities.

## 1 Introduction

Lipopolysaccharide-responsive beige-like anchor protein (LRBA) gene variants leading to Inborn Errors of Immunity (IEI) (1) are clinically rare. To date, only a little over two hundred cases have been reported worldwide, categorizing it as an autosomal recessive inherited disease. Patients with LRBA gene variants primarily exhibit clinical manifestations including hypogammaglobulinemia, recurrent infections (especially respiratory tract infections), increased susceptibility to autoimmune diseases and inflammatory bowel disease (2–4). Subsequent reports have further expanded the clinical phenotype of this disease (4, 5), identifying it as a clinical syndrome with a wide range of variable manifestations.

The patient is a 2-year-old boy who presented the Hematology Department of Tianjin Children’s Hospital in May 2023. The primary clinical symptoms were recurrent respiratory infections and early-onset persistent neutropenia, which subsequently deteriorated into pancytopenia. To further investigate the etiology, we performed whole-exome sequencing on the child and genetic verification on the parents. The results confirmed that the LRBA gene variant carried by the child was a homozygous nonsense variant caused by uniparental disomy of paternal origin, and this variant site has not been previously reported in the medical literature. This report provides a detailed analysis and discussion of the child’s clinical characteristics and genetic data.

## 2 Clinical data

A 2-year-old boy was admitted to the hospital in May 2023 with an 8-day history of fever and cough. His birth history was unremarkable, with age-appropriate growth and development, and no history of chronic diarrhea. Past medical history: Neutropenia was detected when the child was 5 months old, and he had received antibiotic treatment for recurrent respiratory infections. Immunization history: Vaccinations were administered as scheduled, including BCG, polio, measles, DTP (Diphtheria, Tetanus, Pertussis), and hepatitis B vaccines. Family history: The parents are non-consanguineous; the father is a chef and is healthy with no history of recurrent infections or immune dysregulation; the mother is a teacher and is healthy. He has a healthy 6-year-old sister.

Twenty days prior to admission, the patient presented with fever and routine blood test revealed neutropenia and thrombocytopenia (platelets: 50×10^9/L). Physical examination at admission: Normal development and moderate nutrition, no petechiae or purpura on the skin, no significant enlargement of superficial lymph nodes. No enlargement or exudation of bilateral tonsils. Rales and wheezing sounds heard in both lungs. Normal findings in cardiac and abdominal examination, free limb movement, and no abnormalities detected in the neurological examination. Chest and abdominal CT: Scattered inflammatory consolidation in both lungs; multiple lymph nodes with some enlargement in both axillary regions, splenomegaly, and multiple lymph nodes with some enlargement in the abdominal and retroperitoneal areas. Bone marrow aspiration: active proliferation of bone marrow cells, prominent granulocytic hyperplasia, and increased cytoplasmic granules in some granulocytes. 217 megakaryocytes observed with no abnormal morphology. Flow cytometry phenotype of bone marrow cells: No significant abnormal immunophenotypic cells observed. Bone marrow cell cytogenetics are: 46, XY [20]. Pathogen testing: Positive for Mycoplasma pneumoniae IgM, negative for human parvovirus B19 (HPVB19) IgM and IgG, and negative for EB virus and cytomegalovirus DNA. Related hematological and immunological tests are shown in **Table 1**.

**Table 1:** Peripheral blood and immunological data of the patient.

Parameters	Patient values	Reference values
HGB (g/L)	101-107	112-149
WBC (10^9/L)	3.97-6.26	4.40-11.9
NEUT (10^9/L)	0.31-0.57	1.20-7.00
LYMPH (10^9/L)	2.39-4.05	1.80-6.30
PLT (10^9/L)	4-74	188-472
RET (10^12/L)	0.097-0.130	0.024-0.084
IgG (g/L)	7.38	4.53-9.16
IgA (g/L)	0.69	0.20-1.00
IgM (g/L)	0.28	0.19-1.46
IgE (IU/ml)	5.06	0.00-100.00
C3 (g/L)	1.45	0.9-1.8
C4 (g/L)	0.27	0.1-0.4
CD3+ (%)	84.09	53.88-72.87
CD3+ Cell Count (cells/μl)	2889.65	1794-4247
CD3+CD8+ (%)	33.65	19.00-32.51
CD3+CD8+ Cell Count (cells/μl)	1156.26	580-1735
CD3+CD4+ (%)	46.45	24.08-42.52
CD3+CD4+ Cell Count (cells/μl)	1596.08	902-2253
CD16+CD56+ (%)	3.22	7.21-20.90
CD16+CD56+ Cell Count (cells/μl)	110.78	270-1053
CD19+ (%)	11.53	13.23-26.39
CD19+ Cell Count (cells/μl)	396.03	461-1456
IL-2 (pg/ml)	11.11	≤11.4
IL-4 (pg/ml)	5.91	≤5.0
IL-6 (pg/ml)	67.74	≤7.1
IFN-α (pg/ml)	20.54	≤10.5
IFN-γ (pg/ml)	107.8	≤4.5
Regulatory T Cells (%)	4.93	1.2-4.0
Memory Regulatory T Cells (%)	2.8	0.7-2.8
Naive Regulatory T Cells (%)	2.0	0.3-1.9
Activated Regulatory T Cells (%)	1.83	0.04-0.94
ANA	Positive, Cytoplasmic granular pattern, titer 1:80	Negative, titer <1:80
Coombs Test	Negative	Negative

During hospitalization, antibiotics were administered to control the infection. The patient’s temperature normalized after three days, but the platelet count dropped to its lowest at 4×10^9/L, accompanied by petechiae on the skin. Intravenous immunoglobulin (IVIG) at a dose of 1g/kg was given, which increased the platelet count to 74×10^9/L. However, mild anemia and neutropenia persisted.

We conducted whole-exome sequencing for the patient and his parents, with detailed findings as follows:

### 2.1 Methods

Whole exome sequencing (WES) was accomplished by MyGenostics Inc., Beijing, China. Informed consent was signed by the patient’s dependents for genetic analysis, and genomic DNA was extracted from the whole blood taken from the proband and his parents using the QIAamp DNAMini Kit (Qiagen, Shanghai, China) according to the manufacturer instructions. Genomic DNA (1–3 μg) was fragmented to an average size of 150bp by enzyme digestion (MyGenostics Inc., Beijing, China.). Standard libraries including end repair, adapter ligation and PCR amplification were prepared using DNA Sample Prep Reagent Set and exome sequencing library was captured using MGN-ExomeV6 capture kit (MyGenostics Inc., Beijing, China.). The enrichment libraries were sequenced on DNBSEQ-T7 sequencer for paired-reading of 150 bp.

After sequencing, the low quality reads (< 80 bp) were filtered by cutadaptor software (http://code.google.com/p/cutadapt/). The clean reads were mapped to the UCSC hg19 human reference genome using the parameter BWA of Sentieon software. (https://www.sentieon.com/). The variants of SNP and InDel were detected by the parameter driver of Sentieon software(https://www.sentieon.com/) and annotated with multiple databases, such as, 1000 genome, ESP6500, dbSNP, EXAC, Inhouse (MyGenostics), HGMD, and also predicted by SIFT, PolyPhen-2, VariantTaster, GERP++ by ANNOVAR software (http://annovar.openbioinformatics.org/en/latest/) (6). The pathogenicity of variants was assessed following the American College of Medical Genetics and Genomics guideline (ACMG) (7) by assive-sample AI based Causual-variant Evaluation System (MACES)(MyGenostics Inc., Beijing, China).

Then, CNVkit (https://cnvkit.readthedocs.io/en/stable/) software was used to obtain copy number variation information. Futhermore, we identified regions affected by loss of heterozygosity using UPDio (https://github.com/findingdan/UPDio).

### 2.2 Results

**Figure 1:**
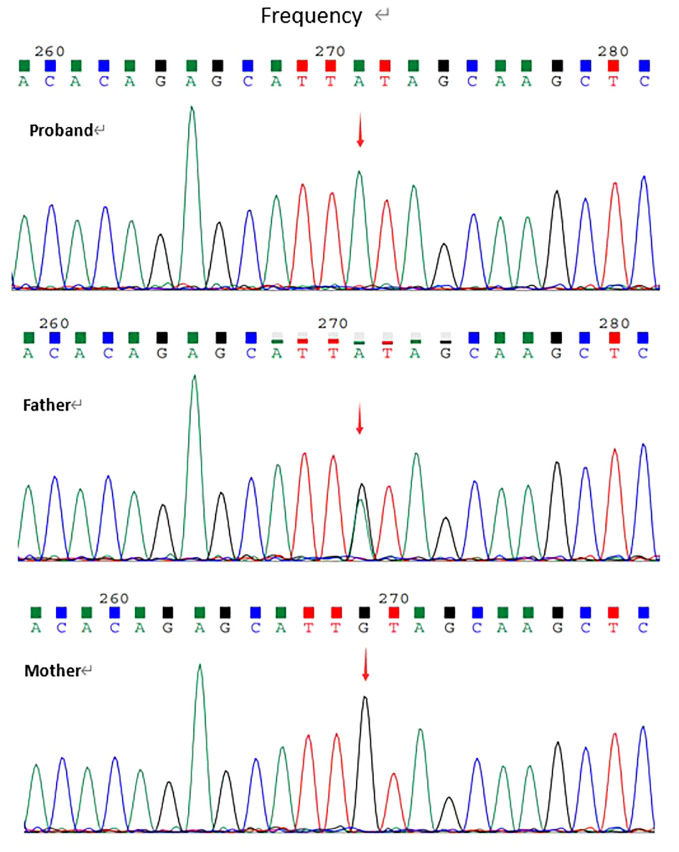
The validation for c.2584C>T(p.Gln862Ter) by Sanger.

Whole exome sequencing revealed a homozygous nonsense variation c.2584C>T(p.Gln862Ter) in exon22 of LRBA gene(NM_006726.4). This variant had not been reported in the literature or databases(HGMD/ClinVar). According to ACMG guidelines, the variation was determined to be Likely pathogenic(PVS1+PM2_supporting). The variation was confirmed by Sanger sequencing for proband and his parents. Sanger data from the parents showed that the patient's healthy father was heterozygous for the variant and his mother did not carry the variant (**Figure 1**).

**Figure 2:**
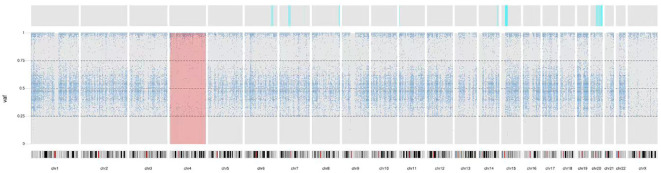
The result of UPD. Homozygous variants on chromosome 4 were>90%. VAF, Variant Allel Frequency.

The WES data of proband showed a loss of heterozygosity (LOH) region of chromosome 4 including the LRBA gene using UPDio, and CNVkit analysis revealed no deletion regions in chromosome 4 (**Figure 2**). Collectively, these results indicate that the homozygous state of the c.2584C>T(p.Gln862Ter) in LRBA variant was due to a UPD4.

## 3 Discussion

The LRBA gene is located at 4q31.3 in the human genome and consists of 58 exons. It encodes a protein of 2863 amino acids with an approximate molecular weight of 319 kDa, and comprises multiple structurally and functionally significant domains. The BEACH domain (beige and Chediak-Higashi) is the most characterized. This domain, with 280 amino acids in length, is conserved in several proteins grouped overall as the BEACH domain containing proteins (BDCP), and is mostly located towards the C-terminal. LRBA protein is relatively highly expressed in lymphocytes and plays a crucial role in the vesicular transport mechanism of immune-related molecules. LRBA protein interacts with the cytoplasmic tail of Cytotoxic T-Lymphocyte Antigen-4 (CTLA-4) by competitively binding with Adaptor Protein-1 (AP-1), thus affecting the stability of CTLA-4. This interaction inhibits the degradation of CTLA-4 in lysosomes (8). CTLA-4 is a key immunoregulatory molecule, primarily expressed on the surface of activated CD4+ and CD8+ T cells, as well as T regulatory (Treg) cells, playing a vital role in the suppressive function of Treg cells. Treg cells are essential for maintaining self-antigen immune tolerance and suppressing excessive immune responses (9). Deficiency in LRBA leads to reduced CTLA-4 expression on the surface of activated T cells and Treg cells. Additionally, LRBA is also a molecule involved in autophagy and participates in apoptosis.

The loss of function of the LRBA gene can lead to a rare autosomal recessive inborn error of immunity disorder, first reported in 2012 by Lopez-Herrera et al. Their research revealed a correlation between pathogenic variants in the LRBA gene and the occurrence of immunodeficiencies and autoimmune syndromes (2). With the continuous advancement in genetic testing technologies and a deepening understanding of this disease in the field of clinical medicine, approximately 212 cases of LRBA deficiency patients have been reported globally as of 2021 (10). These cases involve various types of genetic variants and variant sites, with new types of variants being continually identified.

LRBA deficiency, as an inborn error of immunity disease, is a manifestation of Common Variable Immunodeficiency (CVID). Clinically, it is mainly characterized by hypogammaglobulinemia, recurrent infections in infancy and early childhood (particularly respiratory tract infections), autoimmune diseases (such as immune thrombocytopenia and autoimmune hemolytic anemia), and increased susceptibility to inflammatory bowel disease. However, subsequent reports have expanded the clinical phenotype of this disease, and the clinical phenotype of LRBA deficiency patients is highly variable. It has been reported (11) in a case involving three siblings within the same family, where two of them developed symptoms at 1.5 years and 6 years of age, respectively. In contrast, the third sibling, at 7 years of age (and followed for 2 years), exhibited a homozygous LRBA gene variant but no clinical symptoms. This indicates that even within the same family, the relationship between genotype and phenotype is not entirely clear and may also be influenced by epigenetic or environmental factors.

Uniparental Disomy (UPD) is a concept first proposed by Engel in 1980 (12), referring to an individual having both homologous chromosomes derived from a single parent (in this case, the patient’s both chromosome 4s are from the father). It represents an abnormal combination of homologous chromosomes, deviating from the traditional Mendelian inheritance patterns (13). UPD may lead to abnormal expression of imprinted genes, thereby triggering diseases. With advancements in diagnostic techniques, an increasing number of UPD cases have been identified. The incidence of UPD in newborns is about 1 in 3500 or higher (14), with maternal uniparental disomy (mUPD) being more common than paternal uniparental disomy (pUPD), with a ratio of approximately 3:1. Since chromosome 4 does not have clearly disease-associated imprinted genes, most phenotypes of UPD (4) are primarily caused by the manifestation of recessive genes. Reports of Inborn Errors of Immunity (IEI) caused by UPD are extremely rare (10, 15). In 2018, Spanish researchers reported the first case of a novel homozygous LRBA gene variant caused by mUPD (16), revealing this rare type of genetic variation. This case is the second report of a novel homozygous LRBA gene variant caused by UPD, and it is the rarer form of pUPD. In China, reports of LRBA gene variants are scarce, and there is a lack of systematic research (17, 18).

The Mycoplasma pneumoniae is a common pathogen leading to pulmonary infections in patients with Common Variable Immunodeficiency (CVID) (19). Apart from direct damage, Mycoplasma pneumoniae can induce activation of the host humoral and cellular immune responses, leading to an elevation in various cytokine levels. Mycoplasma antigens can also mimic host cell components, thus the host immune response induced by the pathogen causes auto-immune responses and injuries to multiple organs. Mycoplasma pneumoniae can activate B lymphocytes to produce nonspecific polyclonal antibodies, and some patients with Mycoplasma pneumoniae infection may exhibit weakly positive antinuclear antibodies (ANA). Autoimmune hemolytic anemia can occur in patients with Mycoplasma pneumoniae infection, but pancytopenia is rare (20). In this case, the patient began to exhibit chronic neutropenia and recurrent respiratory infections from the age of 5 months, eventually leading to hospitalization for pancytopenia. Despite effective treatment for infection, the hemocyte profile showed no significant improvement. Bone marrow examination ruled out primary hematological diseases and congenital bone marrow failure syndromes, indicating that the hematological symptoms were also clinical manifestations of LRBA deficiency. Although literature reports that LRBA deficiency patients can present with neutropenia and thrombocytopenia (17), cases with pancytopenia are rare. Whole-exome sequencing of the patient revealed a homozygous variant at c.2584C>T (p.Gln862Ter) in exon 22 of the LRBA gene, predicted to cause a premature stop codon leading to the loss of over 2000 amino acids in the C-terminus of the LRBA protein, including the BEACH domain, resulting in functional loss of the LRBA protein. The patient’s peripheral blood showed reduced numbers of B cells and NK cells, which might be related to increased susceptibility to apoptosis due to autophagy defects (21). T cell counts and IgG levels were normal, consistent with literature reports (4, 5). Normal Treg cell counts might explain the absence of chronic diarrhea and other inflammatory bowel disease manifestations in the patient (4). Imaging studies indicated splenomegaly and enlarged lymph nodes in the axillary and abdominal regions, and the cytokine profile with elevated levels of IFN-α and IFN-γ suggested lymphocyte proliferation and T cell activation. During this course, the patient had concurrent Mycoplasma pneumoniae infection, and elevated levels of IL-6, IFN-α, and IFN-γ were observed. Studies suggest that (22) IL-6 and IFN-α may increase to varying degrees during the acute phase of Mycoplasma pneumoniae infection and decrease in the later stages, while the predictive value of IFN-γ for Mycoplasma pneumoniae infection is currently unclear. Unfortunately, in this case, cytokine levels were not monitored in the later stages of the illness, making it difficult to determine whether the elevated cytokines were due to Mycoplasma infection only or part of the LRBA deficiency. Overall, the clinical symptoms displayed by the patient are currently milder and not as comprehensive compared to typical LRBA deficiency, which could be related to the patient’s age at presentation and the specific pathogenicity of the genetic variant site.

Currently, the treatment for LRBA deficiency primarily relies on immunoglobulin replacement therapy or immunomodulatory agents, including corticosteroids, cyclosporine, mycophenolate mofetil, and rituximab. In 2015, Bernice et al. (8) first used abatacept to treat LRBA deficiency and achieved good therapeutic outcomes. Hematopoietic Stem Cell Transplantation (HSCT) is a crucial treatment modality for patients with Inborn Errors of Immunity (IEI) (5, 23, 24), particularly suitable for patients with complex and severe clinical manifestations. Considering the patient’s age and the lack of involvement of other systems besides the hematological system, the family opted for observation of disease progression and declined further immunotherapy or targeted therapy. However, with the progression of the condition, the patient might require treatment with abatacept or HSCT in the future.

LRBA deficiency is a newly identified genetic immunodeficiency disease discovered in the past decade. The number of patients is relatively small, and new genetic variant types continue to be identified. With the continuous advancement of genetic testing technologies, it is expected that more patients will be diagnosed and treated.

## Data availability statement

The data presented in the study are deposited in the Genome Sequence Archive (Genomics, Proteomics & Bioinformatics 2021) in National Genomics Data Center (Nucleic Acids Res 2022), China National Center for Bioinformation / Beijing Institute of Genomics, Chinese Academy of Sciences repository, accession number is HRA006319.

## Ethics statement

Ethical approval was not required for the studies involving humans because there is no need in accordance with Tianjin Children’s Hospital Ethics Committee. The studies were conducted in accordance with the local legislation and institutional requirements. The participants provided their written informed consent to participate in this study. Written informed consent was obtained from the participant/patient(s) for the publication of this case report.

## Author contributions

LJ: Data curation, Resources, Writing – original draft. SC: Data curation, Resources, Writing – review & editing.
